# An abnormally displaced scaphoid fracture: a case report

**DOI:** 10.1186/1757-1626-2-9309

**Published:** 2009-12-11

**Authors:** Serdar Toker, Volkan Kilincoglu

**Affiliations:** 1 Department of Orthopaedics and Traumatology, Dumlupinar University School of Medicine, 43270 Kutahya, Turkey

## Abstract

Scaphoid fractures are generally known to be difficult to diagnose and difficult to heal. In some reports, up to 40% of scaphoid fractures were reported to be missed at initial presentation. Clinical examinations and plain radiographs are generally poor at identifying scaphoid fractures immediately after injury. In this study we report a scaphoid fracture that has no difficulty in diagnosis because of a very strange and abnormal displacement.

## Introduction

Scaphoid Fractures are relatively common injuries. Differentiation between stable and unstable fractures is always not possible with conventional radiographs and should also be evaluated by computed tomographic scan [1]. Also the scaphoid fractures have the highest prevalence of nonunion in the human body [2]. Welling et al [3], stated that thirty percent of wrist fractures were not prospectively diagnosed on radiography, suggesting that CT should be considered after a negative radiographic finding if clinically warranted. The location of a dorsal scaphoid avulsion fracture emphasizes the need for specific radiographic views or cross-sectional imaging for diagnosis. Scaphoid fracture can be treated by both nonoperatively and surgically. Traditionally, acute nondisplaced scaphoid fractures have been treated nonoperatively in a cast, and the expected union rate approaches 90%. Internal fixation of nondisplaced scaphoid fractures has increased in popularity, and a union rate of 100% has been reported. The growing trend is to recommend internal fixation for the majority of acute scaphoid fractures [4]. In this study we report a scaphoid fracture of which one of the fragments has been banished to distal 1/3 volar side of forearm so there was no difficulty in diagnosis of a scaphoid fracture because of this very strange and abnormal displacement. We could not find such an interesting displacement of scaphoid fracture in the literature.

## Case presentation

**Figure 1:**
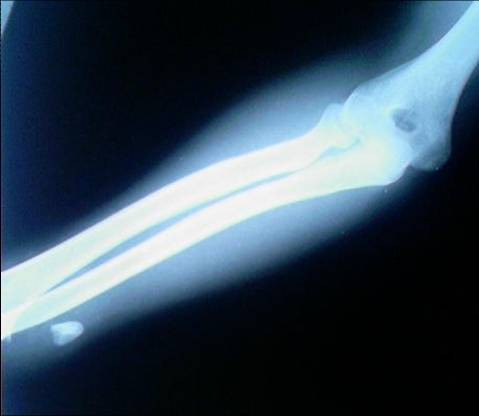
**AP radigraph of elbow and forearm showing a piece of bone at distal 1/3 volar side of forearm**.

**Figure 2:**
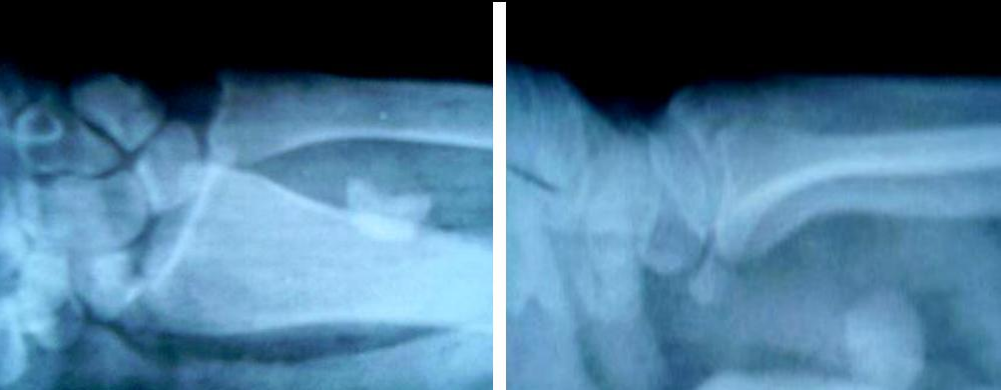
**AP and lateral radiograph of the wrist showing half of the scaphoid at its original location with tha other half placed proximally**.

**Figure 3:**
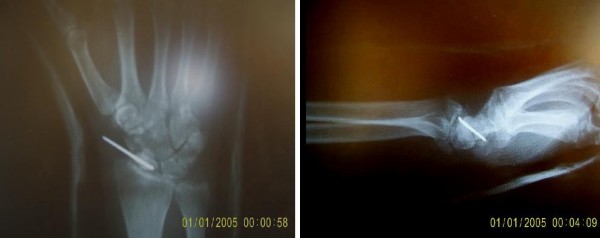
**Postoperative AP and lateral radiograph of the wrist**.

A 32 year-old male from Turkey referred to emergency room after a fall from height on stretched right wrist. Pain, tenderness and swallowing on the wrist and distal forearm were detected in initial physical examination. Echimosis was also detected at distal 1/3 volar side of the forearm. Plain x-ray radiographs of the wrist and forearm were performed. X-ray radiographs showed a piece of bone at 1/3 distal volar side of forearm (Figure 1). It was difficult to think and diagnose a scaphoid fracture for us unless detecting the second half of the scaphoid bone in its original location(Figure 2). No neurovascular injury was present. Urgent surgical reduction and fixation was offered but the patient did not accept the procedure and left the emergency room. After two weeks, the patient came back for a control to our hospital and the radiographies showed an unsatisfactory fixation of the scaphoid bone which was performed in an other hospital (Figure 3). Decision was made to follow-up the course. As far as we know this is the first case of such a surprising and abnormal displaced scaphoid fracture.

## Discussion

Scaphoid fracture is troublesome in orthopaedic practise. Scaphoid fracture and healing are often difficult to diagnose [5]. Numerous studies were performed for the best diagnosis and treatment. According to Nguyen et al [6], up to 40% of scaphoid fractures are missed at initial presentation as clinical examination and plain radiographs are poor at identifying scaphoid fractures immediately after injury. In their study, an extremely high false-negative rate for plain X-rays was identified and they concluded that the appropriate use of CT at initial fracture clinic attendance with 'clinical scaphoid' leads to an earlier diagnosis. Jenkins et al [7], also stated that clinical examination of suspected scaphoid fracture is sensitive, but not spesific and plain radiographs lack sensitivity. In their study, the prevalence of true fracture was found to be 16% and was associated with male sex and injury playing sport. Magnetic resonance imaging had the best diagnostic performance, with the added benefit of soft tissue evaluation, but was the most expensive option. Muller et al [1], emphasized the difficulty of differentiation of stable and unstable scaphoid fractures with conventional radiographs and they stated that computed tomography scans should be performed. Many studies about the scaphoid fractures in the literature are focused on the diagnosis and appropriate treatment of these fractures but we could not find much for the abnormal displacement of the scaphoid fracture except an isolated dorsal fracture-dislocation of the scaphoid reported by Wanajo et al [8]. They stated that such a fracture-dislocation is extremely rare and they believed the pathomechanics of this injury to have been a flexion and radial deviation with an axial force on the wrist.

In our case, the scaphoid bone is fractured and the proximal fragment was dragged along proximally, nearly 8 cm. far away from its original location. We can not define a mechanism to explain such an abnormal displacement and think that this is the first case representing this kind of a scaphoid fracture.

## Consent

Written informed consent was obtained from the patient for publication of this case report and accompanying images. A copy of the written consent is available for review by the Editor-in-Chief of this journal.

## Competing interests

The authors declare that they have no competing interests.

## Authors' contributions

ST wrote the initial draft of and helped revise the manuscript. VK obtained consent from the patients and helped revise the manuscript. Both authors read and approved the final manuscript.
